# Recent increases in terrestrial carbon uptake at little cost to the water cycle

**DOI:** 10.1038/s41467-017-00114-5

**Published:** 2017-07-24

**Authors:** Lei Cheng, Lu Zhang, Ying-Ping Wang, Josep G. Canadell, Francis H. S. Chiew, Jason Beringer, Longhui Li, Diego G. Miralles, Shilong Piao, Yongqiang Zhang

**Affiliations:** 1 CSIRO Land and Water, Black Mountain, Canberra, ACT 2601 Australia; 2 CSIRO Oceans and Atmosphere, PMB #1, Aspendale, VIC 3195 Australia; 3 Global Carbon Project, CSIRO Oceans and Atmosphere, GPO Box 3023, Canberra, ACT 2601 Australia; 4 School of Agriculture and Environment, The University of Western Australia, Perth, WA 6009 Australia; 5 School of Life Sciences, University of Technology Sydney, Ultimo, NSW 2007 Australia; 6 Laboratory of Hydrology and Water Management, Ghent University, Ghent, 9000 Belgium; 7 Sino-French Institute for Earth System Science, College of Urban and Environmental Sciences, Peking University, Beijing, 100871 China; 8 Key Laboratory of Alpine Ecology and Biodiversity, Institute of Tibetan Plateau Research, Chinese Academy of Sciences, Beijing, 100085 China; 9 Center for Excellence in Tibetan Earth Science, Chinese Academy of Sciences, Beijing, 100085 China

## Abstract

Quantifying the responses of the coupled carbon and water cycles to current global warming and rising atmospheric CO_2_ concentration is crucial for predicting and adapting to climate changes. Here we show that terrestrial carbon uptake (i.e. gross primary production) increased significantly from 1982 to 2011 using a combination of ground-based and remotely sensed land and atmospheric observations. Importantly, we find that the terrestrial carbon uptake increase is not accompanied by a proportional increase in water use (i.e. evapotranspiration) but is largely (about 90%) driven by increased carbon uptake per unit of water use, i.e. water use efficiency. The increased water use efficiency is positively related to rising CO_2_ concentration and increased canopy leaf area index, and negatively influenced by increased vapour pressure deficits. Our findings suggest that rising atmospheric CO_2_ concentration has caused a shift in terrestrial water economics of carbon uptake.

## Abstract (web summary)

The response of the coupled carbon and water cycles to anthropogenic climate change is unclear. Here, the authors show that terrestrial carbon uptake increased significantly from 1982 to 2011 and that this increase is largely driven by increased water-use efficiency, rather than an increase in water use.

## Introduction

Global warming, caused mainly by rising atmospheric CO_2_ concentration (*C*_a_), is expected to accelerate the global water cycle^1^ and reduce terrestrial uptake of CO_2_ (i.e. gross primary production, GPP)^2, 3^. At the same time, rising *C*_a_ has significant fertilization effects^4^ by enhancing terrestrial uptake of CO_2_ and by increasing ecosystem water use efficiency (WUE)^5–8^, i.e. the carbon uptake per unit of water loss (evapotranspiration, *E*), which in turn may affect the hydrological cycle^9–11^. Therefore, quantification of the responses of the water and carbon cycles—which are closely coupled because photosynthetic carbon uptake and transpiration both diffuse through leaf stomata—to global warming and rising *C*_a_ is complex. There is still no consensus on how the coupled water and carbon cycles will be altered under global environmental changes, especially under rising *C*_a_ conditions^9–15^, yet quantification of such carbon–water changes is crucial to our predictive capability of future climate change^13, 16^, water availability^9, 10, 17^, food production and the ability to manage the biosphere for climate mitigation and adaptation^18, 19^.

Ecosystem WUE, or the carbon uptake (GPP) per unit of water loss by ecosystem (*E*), is one of the most important ecosystem functional properties in driving terrestrial carbon and water exchanges with the atmosphere^5, 6, 20^. Theory of leaf WUE, defined as leaf photosynthetic carbon uptake per unit of water loss via transpiration, is relatively advanced, and various analytical models can explain variations of leaf WUE with plant function types and climate including those based on the optimization theory^21, 22^. However, understanding of WUE at the ecosystem scale and global levels, defined as GPP per unit ecosystem water loss via *E*, is still very limited^6, 14, 20, 23–25^. This is because other factors, such as soil, forest demography, nutrients and atmospheric feedbacks, come into play to influence ecosystem carbon uptake, water use or both^1, 10, 14^. At present, analysis of ecosystem WUE at regional or global scale usually relies on complex land surface models (LSM) to estimate GPP and *E* in prior^14, 23^, both of which are associated with many poorly quantified interactions across different spatiotemporal scales^14, 26^. Ecosystem GPP at large spatial and temporal scales is unobservable and is not well constrained^25, 27–29^. On the contrary, ecosystem water use, i.e. *E*, can be better constrained with widely available hydrological observations (including precipitation and streamflow) at catchment, regional and global scales, where direct measurements of *E* are not available.

Here we develop a new WUE model independently from GPP and *E*, thus providing a robust alternative to existing WUE products, which in turn enables us to estimate GPP constrained with hydrological measurements. The new analytical WUE model for global terrestrial ecosystems is developed by upscaling leaf WUE directly. Our model takes into account the controls of atmospheric vapour pressure deficit (*D*), *C*_a_ and physiological functioning on leaf WUE. It also partitions among three different components of ecosystem water use through leaf area index (*L*) and the ratio of interception to total water use, i.e. ${f_{{E_{\rm{i}}}}}$ (see Methods). The control of physiological functioning on leaf WUE is accounted for via parameter *g*_1_, which is a physiological parameter related to the functioning and response of stomatal conductance to environmental changes^22, 30^. Unlike previous studies on WUE (e.g. refs. ^6, 20, 23^), this analytical model upscales WUE from leaf to ecosystem directly and reduces uncertainty considerably by not requiring prior estimates of GPP and *E*. Thus, hydrological estimates of ecosystem water use (i.e. *E*) are then used to constrain the estimate of global GPP by multiplying WUE and *E* (denoted as WEC method, see Methods). Trends in the estimate of global WUE and GPP can then be attributed to different drivers analytically. By combining multiple ground-based and remotely sensed land and atmospheric observations into our method, here we show that terrestrial GPP increased by 0.83 ± 0.26 Pg C per year^2^ from 1982 to 2011. Importantly, we find that the GPP increase takes place at no cost of a significant increase in *E*, but is largely (about 90%) driven by the increased WUE, which, in turn, is driven by rising *C*_a_ and enhanced *L*.

## Results

### The validity of the analytical WUE model

**Fig. 1:**
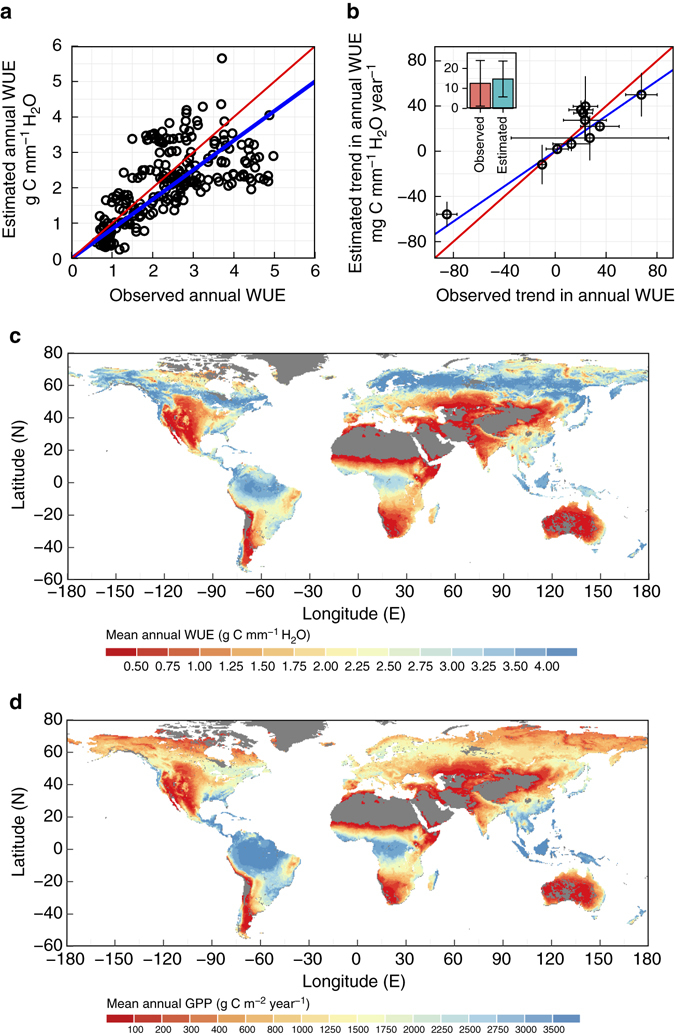
Validation of the ecosystem water use efficiency (WUE) model and spatial variability of estimated mean annual WUE and gross primary production (GPP) over the period of 1982–2011. **a** Validation of annual WUE in g C mm^−1^ H_2_O using observations from 51 eddy covariance flux sites (*n* = 229 site-years). The *red line* is the 1:1 line and *blue line* is fitted using least square regression. **b** Validation of the trends in annual WUE in mg C mm^−1^ H_2_O per year at 11 eddy covariance flux sites with at least 7 years observations. The *red line* is the 1:1 line. The error bars indicate one standard deviation of estimated and observed trends using different methods. The *inset* shows the mean of observed and estimated trends of all the 11 stations. **c**, **d** Estimated spatial details (0.5° × 0.5°) of the global mean annual ecosystem WUE in g C mm^−1^ H_2_O and gross primary production (GPP) in g C m^−2^ per year, respectively, with bare land coloured in grey

The validity of the analytical WUE model is supported by observed ecosystem WUE from eddy covariance sites (see Methods and Supplementary Table 3). The linear correlation between observed and estimated ecosystem WUE of 229 site-years is about 0.64, with a relative bias of −10.6% and mean error of −0.26 g C mm^−1^ H_2_O (Fig. 1a). The slope of the regressed line between observed and estimated annual WUE passing through the origin is 0.84, with an adjusted *R* ^2^ of 0.87. Across 11 sites with more than 6 years of continuous observations, the estimated mean trend in ecosystem WUE is about 14.7 ± 9.0 mg C mm^−1^ H_2_O per year (mean ± 1 standard error), which is consistent with the in situ observed mean trend of 12.6 ± 11.4 mg C mm^−1^ H_2_O per year and with previous findings^23^. The linear correlation coefficient between observed and estimated trends in annual WUE is about 0.93, with a relative bias of 17.2% and mean error of 2.16 mg C mm^−1^ H_2_O per year (Fig. 1b). The slope of the regressed line between observed and estimated annual WUE passing through the origin is 0.78, with an adjusted *R* ^2^ of 0.85.

We also assess the validity of our analytic model at the global scale by comparing the estimated global mean and spatial variation of ecosystem WUE with other independent estimates. To account for input uncertainties, we used 10 different ground-based and remotely sensed observations of global land and atmospheric forcing, and obtained an ensemble of 12 estimates of global WUE with a spatial resolution of 0.5° by 0.5° (see Methods). We estimate the mean annual global WUE of 12 estimates to be 2.1 ± 0.35 g C mm^−1^ H_2_O (mean ± 1 standard deviation, hereafter the uncertainty is expressed as 1 standard deviation unless specified) from 1982 to 2011. Our global mean annual estimate is close to, but about 20% larger than, the ensemble mean of the six LSMs (Supplementary Table 4) of 1.66 ± 0.26 g C mm^−1^ H_2_O, and the independent estimates using the model tree ensemble (MTE) data set^31^ of 1.66 ± 0.02 g C mm^−1^ H_2_O. Our estimated WUE shows distinctly spatial and latitudinal patterns (Fig. 1c), with low values in the arid regions and high values in the tropical and boreal forests. Along different latitudes, it is relatively high in the northern high latitude around 60°N and in the tropical region around 3°S. This spatial pattern of estimated global WUE is also consistent with other independent estimates (see Supplementary Fig. 4).

### Estimated global GPP

Based on the 12 annual WUE estimates and 7 independent *E* products (see Methods and Supplementary Table 2), we obtain an ensemble of 84 estimates of global annual GPP from 1982 to 2011 by multiplying WUE and *E* (WEC method, see Methods). The estimated mean annual global GPP of 84 estimates is 146.1 ± 21.3 Pg C per year, and falls within the reported range of global GPP from 90 to 210 Pg C per year^27, 29, 32^. The spatial details of our GPP estimates accord well with estimates derived using the MTE method or LSMs at the global scale (Supplementary Fig. 5). This provides further confidence that the new WUE model represents a robust and quantitative measure of the functional coupling between the terrestrial water and carbon cycles, specifically GPP and *E*, allowing the use of hydrological observations to constrain GPP estimates and explaining the causality of the estimated trends in GPP over the recent years.

### Trends in global GPP and WUE and their attribution

**Fig. 2:**
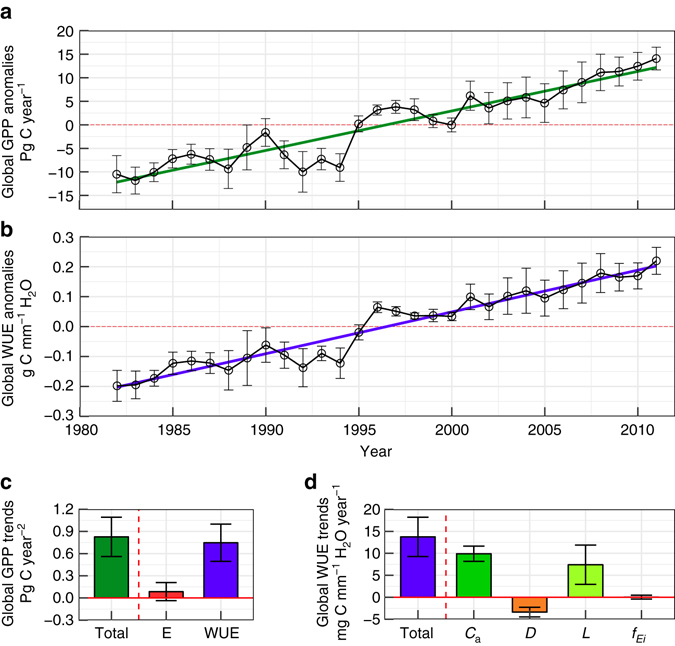
Estimated trends in global gross primary production (GPP) and water use efficiency (WUE) and their drivers over 1982–2011. **a**, **b** Annual mean anomalies and its standard deviation (shown as error bars) of global GPP in Pg C per year (*n* = 84) and global WUE in g C mm^−1^ H_2_O (*n* = 12) during 1982–2011, respectively. The *green line* in **a** and *blue line* in **b** represent the linear trend over the past three decades. **c** Contribution of *E* and WUE to total global trends in GPP (Total). **d** Contributions of four factors to the total increase in global WUE (Total). *C*_a_, *D*, *L* and ${{\boldsymbol{f}}_{{{\boldsymbol{E}}_{{\bf i}}}}}$ refer to contributions from atmospheric CO_2_ concentration, vapour pressure deficit, leaf area index and fraction of canopy interception to total ecosystem water use, respectively. The error bars in all the subplots represent one standard deviation

Using the full ensemble of estimates (*n* = 84), we estimate that global GPP has increased 0.83 ± 0.26 Pg C per year^2^ from 1982 to 2011 on average (Fig. 2a, c, *p* < 0.001), or 7.33 ± 2.09 g C m^−2^ per year^2^, or 0.6 ± 0.2% per year of mean annual GPP. By excluding one *L* product that with systemic inconsistency in some regions (see Discussion), our best estimate of the trend in global GPP, based on an ensemble of 42 estimates, is 0.59 ± 0.12 Pg C per year^2^ (0.33–0.87 Pg C per year^2^).

The range of the estimated trend in global GPP over 1982–2011 by the WEC method is 0.33–1.30 Pg C per year^2^, which is much wider than that derived from the six LSMs (0.32–0.57 Pg C per year^2^). Reported values in other studies give a range from 0.2 to 0.66 Pg C per year^2^ (see refs. ^28, 33, 34^). The estimated trend in global GPP from all the 84 GPP estimates is larger than the ensemble of 6 LSMs (0.44 ± 0.08 Pg C per year^2^) over the same period. However, our best estimate of 0.59 ± 0.12 Pg C per year^2^ (*n* = 42) is quite close to the LSMs mean ensemble.

All seven *E* data sets show small trends over 1982−2011 with one being negative, two being not significantly different from zero (*p* > 0.05) and other four being significantly positive (*p* < 0.05, see Supplementary Fig. 3). The mean trend of all the seven *E* data sets is 0.37 ± 0.80 (range from −1.1 to 1.8) mm per year^2^, or 0.06 ± 0.13% per year of mean annual *E*. We estimate global ecosystem WUE has increased at a mean rate of 13.7 ± 4.3 mg C mm^−1^ H_2_O per year from 1982 to 2011 (Fig. 2b, d, *p* < 0.001), which is about 0.7 ± 0.2% per year of mean annual WUE.

The two drivers (*E* and WUE) contributing to the trends in terrestrial GPP are further investigated (see Methods) and shown in Fig. 2c. Globally, both *E* and WUE positively contribute to the estimated increase in GPP. The contribution of WUE to the estimated GPP trend is about 0.75 ± 0.25 Pg C per year^2^ or 90 ± 30% of the total GPP trend, as compared to about 0.09 ± 0.12 Pg C per year^2^ or 10 ± 15% of the total GPP trend by *E*. The results suggest that estimated increase in global GPP under climate change and rising *C*_a_ conditions over the past 30 years is taking place at no cost of using proportionally more water, but it is largely driven by the increase in carbon uptake per unit of water use, i.e. WUE.

Contributions of the four different variables (i.e. *C*_a_, *D*, *L* and ${f_{{E_{\rm{i}}}}}$) to the estimated trend in WUE are further analysed (see Methods) and shown in Fig. 2d. Three of the four variables (except ${f_{{E_{\rm{i}}}}}$) have significant (*p* < 0.01) contributions to the estimated increasing trend in global WUE, of which *C*_a_ and *L* have positive contributions but *D* has a negative contribution to the estimated increase in WUE. The contributions of *C*_*a*_, *D*, *L* and ${f_{{E_{\rm{i}}}}}$ to the estimated trend in global WUE are 9.9 ± 1.7, −3.4 ± 1.1, 7.4 ± 4.3 and 0.04 ± 0.4 mg C mm^−1^ H_2_O per year, or 77 ± 20%, −27 ± 11%, 49 ± 16% and 0.2 ± 3%, respectively. The contributions of four drivers of WUE (i.e. *C*_a_, *D*, *L* and ${f_{{E_{\rm{i}}}}}$) to the estimated global GPP trends are 0.53 ± 0.06, −0.18 ± 0.04, 0.41 ± 0.24 and 0.003 ± 0.02 Pg C per year^2^, or 64 ± 7%, −21 ± 5%, 49 ± 29% and 0.3 ± 3%, respectively. Therefore, recent changes in *C*_a_, *D* and *L* are not only the main drivers of the estimated global WUE trend, but also estimated global GPP trend.

### Spatial variability of the estimated GPP and WUE trends

**Fig. 3:**
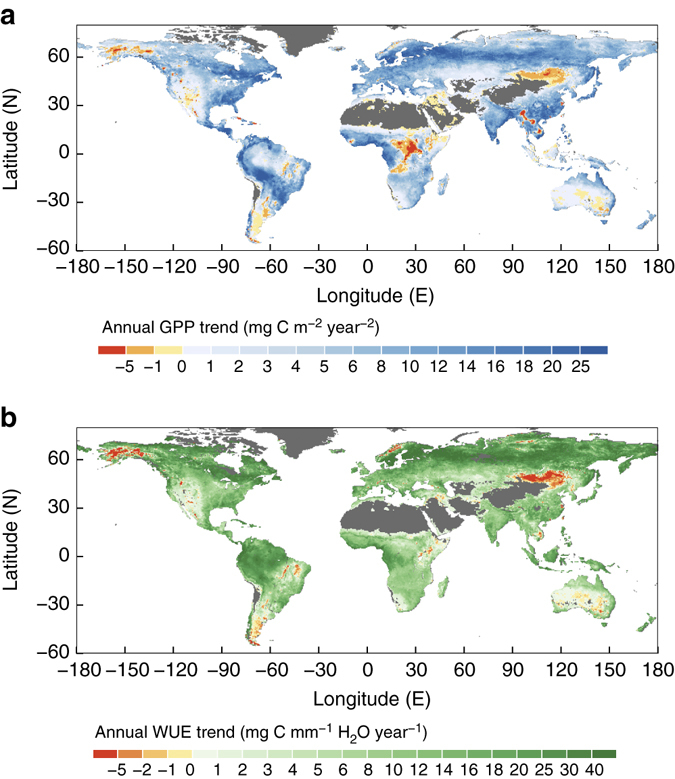
Estimated spatial trends in annual gross primary production and water use efficiency over 1982–2011. **a** Spatial variation (0.5° × 0.5°) of the linear trend in ecosystem GPP in g C m^−2^ per year^2^. **b** Spatial variation (0.5° × 0.5°) of the linear trend in ecosystem WUE in mg C mm^−1^ H_2_O per year

Spatially the trends of estimated GPP and WUE over the period of 1982−2011 are quite variable (Fig. 3). The GPP trend varies from −4.4 to 27.2 g C m^−2^ per year^2^ (1−99% range, Fig. 3a). Despite the large-scale occurrence of droughts and disturbances over the study period^35^, remarkably, about 82 ± 5% of vegetated land shows positive trends in GPP, of which about 80 ± 8% of the trends is significant (*p* < 0.05). About 55 ± 11% of vegetated areas show positive trends in *E*, of which only about 47 ± 4% is significant (*p* < 0.05). Changes in WUE range from less than −6.6 to 51.3 mg C mm^−1^ H_2_O per year (1−99% range, Fig. 3b). An increase in ecosystem WUE is found in 90 ± 2% of vegetated areas over 1982–2011, of which about 78 ± 8% is significant (*p* < 0.05). The remaining 10 ± 2% of the vegetated land shows a decreasing trend, of which only about 21 ± 7% is significant (*p* < 0.05).

**Fig. 4:**
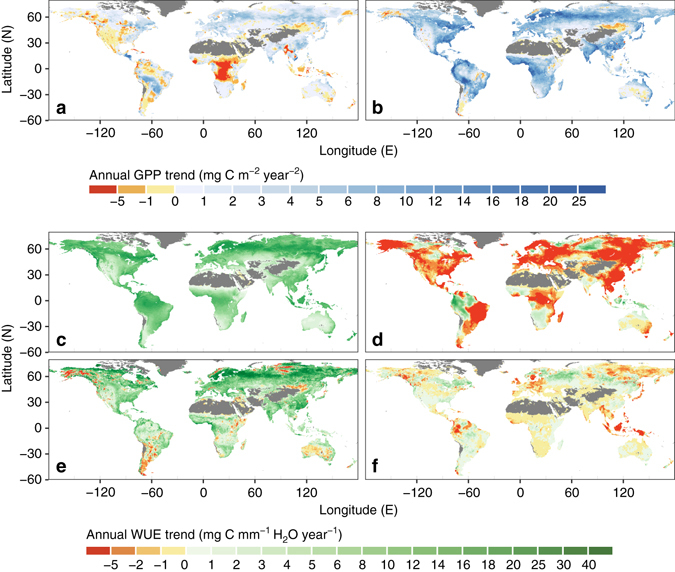
Estimated spatial variations of the contributions to trends of ecosystem gross primary production (GPP) and water use efficiency (WUE) from different variables over 1982–2011. **a**, **b** Spatial details of the contributions to recent trend in GPP from ecosystem water use and WUE in g C m^−2^ per year^2^, respectively. **c**–**f** Spatial details of the contributions to trends in ecosystem WUE atmospheric CO_2_ concentration **c**, vapour pressure deficit **d**, leaf area index **e** and fraction of canopy interception in mg C mm^−1^ H_2_O per year **f**

Generally, spatial patterns of GPP and WUE trends are similar (Fig. 3a, b), which further supports that WUE is the dominant contributor to the estimated GPP trend in most regions. Regions with large increases in GPP and WUE are mainly in boreal and tropical forests. Negative trends in both WUE and GPP are found only over small areas in northeast China and Mongolia, and the inland of Australia. Central Africa experienced a decrease in GPP (Fig. 3a) but an increase in WUE (Fig. 3b), which is associated with a significant declining *E* trend in this region (Fig. 4a) outweighing the increase in WUE.

### Contributions from different drivers

Contributions of WUE and *E* to the estimated GPP trend at global level (Fig. 4a, b) are both positive, although they can be negative or positive regionally at grid level. Regions with negative contributions from either WUE or *E* are limited. Only about 12 ± 5% and 3 ± 1% of regions have significant negative contributions (*p* < 0.05) from *E* and WUE, respectively. Significant positive contribution of *E* (*p* < 0.05) to the estimated GPP trend is found in about 27 ± 4% of the vegetated land, whereas significant positive contribution of WUE (*p* < 0.05) is found in about 66 ± 9% of vegetated land. Positive contributions of *E* are found mainly in the east of North America, boreal forest regions in Europe and Asia, and in the southeast of China (Fig. 4a). Positive contributions of WUE are found in most regions globally but negative contributions of WUE are limited to some small patches in the Northeast of China, Mongolia and inland of Australia (Fig. 4b).

The contributions of four different variables to the estimated trend in WUE at the grid level are shown in Fig. 4c–f. The *C*_a_ has positive effects on ecosystem WUE globally as it is related linearly with WUE over all vegetated land and has increased monotonically over the study period. For the other three variables, their contributions vary spatially from negative to positive. Changes in *D* have negative effects on ecosystem WUE in about 69 ± 3% of the vegetated land area, especially in Europe, East Asia, Alaska and the eastern parts of South America, and have positive effects in the northwest of South America, the north of central Russia, and the south of Africa. However, vapour pressure deficit (*D*) has significant (*p* < 0.05) negative effects on WUE only in about 37 ± 2% of the global vegetated area and significant (*p* < 0.05) positive effects only in about 10 ± 3% of the global vegetated area. Changes in *L* have significant (*p* < 0.05) positive effects on WUE in about 52 ± 17% of the vegetated areas, especially at northern high latitudes, and have significant negative effects only in a small fraction of vegetated land (~4 ± 1%). Changes in ${f_{{E_{\rm{i}}}}}$ have only marginal contribution to the global changes in WUE because the magnitude of the contribution is small (Fig. 2d) and extent of the significant contribution is very limited (~18%).

## Discussion

The carbon uptake capacity of terrestrial ecosystems is both determined by availability of radiation (or light) and water^27, 36, 37^. At large scales (>100 km^2^), GPP cannot be directly observed and is typically estimated using remotely sensed products, LSMs or ecosystem models^36^ with many poorly constrained interactions and assumptions^14, 25, 26, 29^. There is therefore considerable uncertainty in GPP estimates^27, 29, 38^. Although the coupling of plant carbon uptake and water use is widely recognized^20, 27, 28^, ecosystem water use has not been widely used to constrain GPP at regional or global scales at present^27, 37^. Here we provide a promising new way to constrain GPP with ecosystem water use (*E*) by taking advantage of two merits of the proposed methodology. One is that ecosystem WUE is defined in terms of ecosystem total *E* rather than transpiration, which enables us to directly utilize widely available field measurements and hydrological estimates of *E*. This is because (1) different components of *E* are rarely measured separately and (2) total *E* is a critical component in the water cycle and is routinely reported in hydrological observations and studies. The other advantage is that ecosystem WUE in this study is estimated without prior knowledge of GPP or *E*, which enables us to use hydrological estimates of *E*, such as from catchment water balance studies, to constrain GPP. In this study, *E* data sets, which are well validated against global hydrological observations (including rainfall and streamflow data) and eddy flux measurements, are used as surrogate of hydrological observations to constrain the estimates of GPP. The good agreement of the spatiotemporal variability of the estimated GPP using the WEC method with other independent products (see Fig. 1d and Supplementary Fig. 5) suggests that our approach provides a functional and robust quantification of the coupling between terrestrial water and carbon cycles and allows us to constrain ecosystem GPP using widely available hydrological observations.

Global *E* data sets show that *E* increased little during 1982–2011 at about 2% of the mean annual *E*, while global GPP increased significantly at about 17% of the mean annual GPP over the study period. This disproportionate change in the coupled water and carbon cycles globally indicates a shift in water-use economics of terrestrial carbon uptake as *C*_a_ rise, and that the increase in terrestrial GPP had little quantitative consequences in the ecosystem total water use. However, there are some impact on the water cycle at regional scales as we showed in our spatially explicit trend analysis (Figs. 3 and 4) or in hydrological partitioning^9^. Regional changes in water cycle depend on the direct water-saving effects of rising *C*_a_ by suppressing vegetation transpiration, and on some indirect effects of rising *C*_a_ that can offset or outweigh the water-saving effects, such as (1) increased in leaf temperature and *D* locally due to reduced transpiration, (2) increased in *L* resulting from the fertilization effects on GPP and (3) increased in *D* globally due to global warming.

Current understanding about the change in global GPP is still very limited and depends largely on earth system models with significant uncertainties^3, 14, 27, 39^. Here we provide a new way to estimate changes in global GPP and our estimated global trend in GPP is comparable with that derived from LSMs and independent studies. Across different eco-regions, trends in GPP estimated by the WEC method agree well with those derived from six LSMs in term of the positive changes. Mean trends from the WEC methods are close to those from the LSMs at different ecoregions (Supplementary Table 7 and Supplementary Fig. 6), except for the temperate and boreal forests and Tundra regions, where the mean trends estimated using WEC method are larger than those derived from LSMs (see Supplementary Discussion and Supplementary Fig. 7). Basically, current understanding about the trends in GPP at different spatial scales (i.e. from site to regional and global scales) is still very uncertain. Although not a proof, our findings of a significant increase in global GPP are consistent with other studies and with evolution of the global carbon budget over the past 30 years^3, 12^.

Spatially, an estimated large increase in GPP can be found mainly in two regions, i.e. the boreal forest and tropical forest regions (Fig. 3a). The relative large GPP increase in the boreal forest region results from positive contributions from both *E* (Fig. 4a) and WUE (Fig. 4b). In turn, positive contribution of WUE in this region is mainly driven by rising *C*_a_ and enhanced canopy *L* (Fig. 4c, e). For the tropical forest region, the large GPP increase mainly results from the increase in WUE in this region, caused by rising *C*_a_ (Fig. 4c), which is consistent with increased WUE trend derived from tropical tree growth rings^7^.

At the global scale, the most important driver for the increases in GPP and WUE from 1982 to 2011 is rising *C*_a_. Global GPP increases at a rate of 17% per 10% increase in *C*_a_. The rising *C*_a_ is the largest contributor to the estimated trend in GPP and enhances GPP at a rate of about 8% per 10% increase in *C*_a_, which is larger than the observed effect on net primary production from free-air CO_2_ experiments about 5% per 10% increase in *C*_a_^40, 41^. The analytical model estimates that global WUE increases at a rate of 14% per 10% increase in *C*_a_, of which the largest contributor is *C*_a_. This is consistent with estimated global WUE trend and its attributions from LSMs^23^ and with the sensitivity of ecosystem WUE to *C*_a_ obtained from eddy flux observations and long-term tree ring records^24^. Furthermore, the rising *C*_a_ may have an overall bigger role in the global water and carbon cycles than we reported here as increases in *L* can also be attributed to rising *C*_a_^42, 43^.

After rising *C*_a_, the change in *L* is the second largest contributor to the estimated trends of GPP and WUE globally. The impact of change in *L* on GPP is only indirectly accounted for in the data products of *E* and estimated WUE. The two *L* products used in this study show an increasing trend in *L* in the past three decades^42^. Increase in *L* can lead to a positive trend in *E* because ecosystems with a greater *L* typically have higher interception and transpiration^44, 45^. Increase in *L* can have both positive and negative effects on ecosystem WUE in this study. An increase in *L* can increase ecosystem WUE by increasing transpiration fraction of ecosystem water use, or can decrease ecosystem WUE by increasing canopy interception (see Methods). On average, ecosystem WUE increases with *L* to about 3 and then decreases with *L* at the global scale (Supplementary Fig. 8). In reality, however, the controls of *L* on ecosystem water use partitioning are more complex than the method we used in this study^46^^,^^47^. The changes in *L* caused by environmental changes are complex^10, 42, 43, 48^, which poses limits to the capacity for LSMs and the analytical WUE model here to project changes in future water and carbon cycles given that all parameterize *L* as the major control of water, energy and carbon fluxes between vegetated land surface and atmosphere.

One major source of uncertainity in the estimated annual WUE and GPP comes from the input data sets. Uncertainties in the magnitude of global annual GPP is largely sourced from the differences in *D* amongst three data sets and different magnitudes of global *E* between reanalysis and diagnostic products. Global annual *E* from reanalysis data sets is typically much larger than that of diagnostic *E* data sets. Uncertainties in the global GPP trend are primarily sourced from different trends in *E* and *L* products (Fig. 2c, d). The uncertainty in the contribution of *E* to global GPP trend (Fig. 2c) is largely resulted from two *E* data sets that have very different trends from the other five *E* data sets. The uncertainty in the contribution of *L* to global WUE (or GPP) trend results from a spurious step change around 2000 in one *L* product. Trends in global GPP from two *L* products are 0.59 ± 0.12 and 1.06 ± 0.13 Pg C per year^2^, respectively. The differences in *D* amongst three climate forcing data sets do not lead to significant uncertainty in the global WUE (or GPP) trends (Fig. 2d) as trends in *D* amongst three data sets agree with each other well. In summary, differences amongst the input data sets do not alter the estimated increasing trends in WUE and GPP at global scale (Fig. 2a, b), and therefore the conclusion of this study (see Supplementary Discussion).

The success of the developed analytical WUE model highlights the importance of the physiological parameter *g*_1_ for predicting the functional and biogeographical variation in WUE, which implies that plant functional traits are very important to advance our understanding about the coupled water and carbon cycles and their changes (also see refs. ^25, 29^). By directly accounting for key controls, our WUE model is analytically tractable, and lends itself to identifying the major drivers of changes in ecosystem WUE with quantifiable uncertainty. The ecosystem WUE model we have developed uses a top-down approach^49, 50^ that provides a meaningful conceptualization of ecosystem WUE by directly accounting for key controls (or first-order controls) at the annual time scale and neglecting other factors that may become important at shorter (e.g. diurnal to seasonal) or longer (e.g. multiple decades to century) time scales including diurnal variations in meteorological conditions^10^, variable controls of *L* on *E* partitioning, long-term forest demography^4^ and nutrient limitations^40, 51^. Therefore, our model is best fitted for applications at the annual to decadal time scales as a diagnostic tool. The analytical WUE model captures satisfactorily the spatial and temporal variability of observed annual ecosystem WUE and produces a global pattern of WUE consistent with other independent estimates. However, unexplained variability in ecosystem annual WUE and its trends by the proposed model is large at the site level (Fig. 1a, b). Current available flux data are too limited to quantify the magnitude of trends in global WUE and GPP and the associated uncertainty. There are 11 stations with long enough data for trend validation (Fig. 1b) covering seven different plant function types based on our data-selection criteria. The number of stations for every plant function types is no more than 3. There is only one station located in the tropical forest region and no station in the boreal forest regions for trend validation. In addition, the estimated average trend in observed annual WUE has a large uncertainty (Fig. 1b) and is also much lower than that reported by ref. ^6^. It reflects the fact that trends in ecosystem WUE depend on the way it is defined and the study regions and highlights that long-term observations available for detecting changes in global water and carbon cycles are still limited, especially in the tropical and boreal forest regions where we find larger trends in WUE than in other regions. Therefore, further development and validation of the model are needed to reliably interpret ecosystem WUE and its changes at annual and finer spatiotemporal scales.

Our results show that terrestrial GPP has increased significantly and is primarily associated with increase in WUE, which in turn is largely driven by rising *C*_a_ and increase in *L*. Little increase in *E* but significant increase in GPP suggests that increase in terrestrial carbon uptake over the past three decades has had little consequences in global ecosystem water use (but there can be significant changes at regional scale). Across different regions, boreal and tropical forests increased their ecosystem WUE and annual GPP most significantly, with the increase in *L* and *C*_a_ as the major drivers in boreal region and the increase in *C*_a_ as the main driver in the tropical region.

## Methods

### The analytical WUE model

In this study, the ecosystem WUE is defined as ecosystem GPP per unit of ecosystem water loss via evapotranspiration (*E*), i.e.

1 $${\rm{WUE}} = \frac{{{\rm{GPP}}}}{E}$$

In Eq. (1), evapotranspiration, *E*, includes both productive water use, i.e. transpiration (*E*_t_), and non-productive water use, i.e. evaporation from soil surface (*E*_s_) and evaporation from canopy interception (*E*_i_), namely,

2 $$E = {E_{\rm{t}}} + {E_{\rm{s}}} + {E_{\rm{i}}}$$

Productive water use, *E*_t_, is coupled to carbon assimilation as both CO_2_ and water diffuse through leaf stomata. However, non-productive water uses, i.e. *E*_i_ and *E*_s_, are only indirectly related to carbon productive processes. The role of non-productive water use on carbon production represents a major difference between leaf-scale and ecosystem-scale WUE. For growing season when GPP and *E*_t_ are greater than zero, then Eq. (1) can be reformulated as:

3 $${\rm{WUE}} = \frac{{{\rm{GPP}}}}{E} = \frac{{{\rm{GPP}}}}{{{E_{\rm{t}}}}}\frac{{{E_{\rm{t}}}}}{{{E_{\rm{t}}} + {E_{\rm{s}}}}}\left( {1 - \frac{{{E_{\rm{i}}}}}{E}} \right)$$

Equation (3) states that growing season ecosystem WUE can be estimated as the product of three terms. The first is transpiration WUE, i.e. GPP/*E*_t_. The second is partitioning between transpiration and soil evaporation during carbon assimilation period. The last term is one minus the fraction of interception to total water use, *E*_i_/*E* (denoted as ${f_{{E_{\rm{i}}}}}$).

Previous studies have shown that leaf WUE is quite independent of the growth environment^21^ and can be directly scaled to canopy scale^52, 53^. Thus ecosystem transpiration WUE, i.e. GPP/*E*_t_ in Eq. (3), can be approximated by leaf WUE (WUE_l_) as in ref. ^22^:

4 $$\frac{{{\rm{GPP}}}}{{{E_{\rm{t}}}}} = \frac{{\mathop {\int }\nolimits A{\rm{d}}t}}{{\mathop {\int }\nolimits T{\rm{d}}t}} \approx {\rm{WU}}{{\rm{E}}_{\rm{l}}} = \frac{A}{T} = \frac{{{C_{\rm{a}}}{p_{\rm{a}}}}}{{1.6\left( {D + {g_1}\sqrt D } \right)}}$$

where *A* is leaf net photosynthetic carbon uptake (µmol(CO_2_) m^−2^ s^−1^); *T* is leaf transpiration (µmol(H_2_O) m^−2^ s^−1^); *C*_a_ is ambient atmospheric CO_2_ concentration in mol(CO_2_) mol^−1^(air); *g*_1_ is an empirical parameter of the Ball stomatal conductance model^54^ (kPa^0.5^); *D* is the vapour pressure deficit (kPa); and *p*_a_ is atmospheric pressure (kPa). The stomatal conductance model used in Eq. (4) is similar to the Ball–Berry stomatal conductance model that has been widely adopted in most global land surface models. What is different is that parameters in Eq. (4) have meaningful ecological interpretations as stated in ref. ^22^; and were estimated using extensive field observations as provided in ref. ^30^.

Partitioning of transpiration and soil evaporation can be evaluated via Beer’s Law as:

5 $$\frac{{{E_{\rm{t}}}}}{{{E_{\rm{t}}} + {E_{\rm{s}}}}} = 1 - {\rm{exp}}\left( { - kL} \right)$$

where *L* is the canopy leaf area index, and *k* is the radiation extinction coefficient. Partitioning of transpiration and soil evaporation is much more complex than Beer’s law in reality^46^^,^^47^, especially at short times scales (i.e. hourly or diurnal scales). However, this study is focused on monthly to annual time scales, at which Beer’s law can provide reasonable and accurate partitioning between transpiration and soil evaporation^46^^,^^55^^,^^56^.

Therefore, Eq. (3) for ecosystem WUE can be formulated as:

6 $${\rm{WUE}} = \frac{{{C_{\rm{a}}}{p_{\rm{a}}}}}{{1.6\left( {D + {g_1}\sqrt D } \right)}}\left[ {1 - {\rm{exp}}\left( { - kL} \right)} \right]\left( {1 - {f_{{E_{\rm{i}}}}}} \right)$$

If the input of *C*_a_ in Eq. (6) is in ppm (1 ppm ≈ 1 µmol(CO_2_) mol^−1^(air)), estimated WUE by Eq. (6) has a unit of µmol(CO_2_) mol^−1^(H_2_O). It can be converted to g C mm^−1^ H_2_O by a factor of about 0.667 × 10^−3^ (i.e. 1 µmol(CO_2_) mol^−1^(H_2_O) ≈ 0.667 × 10^−3^ g C kg^−1^ H_2_O ≈ 0.667 × 10^−3^ g C mm^−1^ H_2_O).

In this study, Eq. (6) is applied to annual time scale for identifying the major drivers of changes in ecosystem WUE based on the readily available data only. Assuming that GPP and *E*_s_ during the non-growing season is negligible, we use Eq. (6) to estimate ecosystem annual WUE with the first two terms (i.e. $\frac{{{C_{\rm{a}}}{p_{\rm{a}}}}}{{1.6\left( {D + {g_1}\sqrt D } \right)}}$ and $\left[ {1 - {\rm{exp}}\left( { - kL} \right)} \right]$) are estimated during growing season months only and the third term (i.e. $\left( {1 - {f_{{E_{\rm{i}}}}}} \right)$) is estimated at an annual time scale, in which data requirement of Eq. (6) is minimal and seasonal variations in ecosystem WUE are also preserved. We define the growing season as mean monthly air temperature >0 °C (Supplementary Fig. 1 and see Supplementary Discussion for more details).

### Estimating global ecosystem WUE

Two parameters and five variables are required for estimating WUE according to Eq. (6). The two parameters are *k* and *g*_1_. The five variables are *C*_a_, *p*_a_, *D*, *L* and ${f_{{E_i}}}$. Parameter *k*, the extinction coefficient of radiation, is set as a constant of 0.6 for all the vegetated land. Global map of parameter *g*_1_ (Supplementary Fig. 2) is generated by interpolating *g*_1_ values of different plant functional types (Supplementary Table 1) adopted from ref. ^30^ with a global vegetation classification map (i.e. SYNMAP^57^). The data for variable *C*_a_ are observed annual mean data at Mauna Loa site^58^ and accessed from NOAA’s Earth System Research Laboratory. Variable *p*_a_ is a mean annual atmospheric pressure field estimated from the monthly climatology data set developed by Climatic Research Unit (CRU)^59^. Three different inputs for variable *D* are used, which are derived from three global climate forcing data sets including the CRU-NCEP data set^59^, the Princeton Global Forcing data^60^ and the WATCH Forcing Data ERA-Interim (WFDEI) meteorological forcing data^61^. Two global leaf area index data sets, GIMMS-LAI3g^62^ and GLASS-LAI^63^, are used as input for variable *L*. Two different global interception ratios for variable ${f_{{E_{\rm{i}}}}}$ are used, which are derived from the Global Land Evaporation Amsterdam Model (*E*_GLEAM_)^64^ and the CSIRO global evapotranspiration data set (*E*_CSIRO_)^45^. All the spatial data are gridded to a spatial resolution of 0.5° using area weighted local area mean. This study focuses only on vegetated land cells that are defined as having a mean annual Normalized Difference Vegetation Index >0.1 based on the GIMMS NDVI3g data set^65^. Based on 10 widely used atmospheric, land and remotely sensed observations for these five variables, 12 estimates of WUE are derived of an overlapping period from 1982 to 2011.

### Estimating global ecosystem GPP

Based on the analytical WUE model, ecosystem WUE and *E* are multiplied to estimate ecosystem carbon uptake (denoted as WEC method) as,

7 $${\rm{GPP}} = {\rm{WUE}} \times E$$

The proposed WEC model for estimating ecosystem GPP is based on the assumption that WUE is an ecosystem functional property that couples GPP and *E*. Therefore, ecosystem *E* data sets, which can be estimated from global observed hydrological observations, are used to constrain estimation of GPP.

In total, 84 estimates of global annual GPP are obtained by multiplying 12 annual WUE estimates and 7 independent data sets of annual *E* (Supplementary Table 2 and Supplementary Fig. 3). Global *E* data sets are used as a surrogate of hydrological observations to constrain GPP estimation. Seven *E* data sets collected including *E*_MTE_^31, 35^, *E*_GLEAM_^64^, *E*_CSIRO_^45^, *E*_WB-MTE_^66^, *E*_MERRAa_^67^, *E*_MERRAs_^68^, and *E*_ERA_^69^. The first four *E* data sets are diagnostic data sets, mostly based on in situ and satellite remote sensing forcing, while the rest three are re-analysis data sets. The diagnostic *E* data sets have been widely validated against eddy flux measurements and hydrological observations including precipitation and streamflow data. The *E*_MTE_ and *E*_WB-MET_ are estimated using the MTE method from global observed water fluxes at eddy flux sites^31, 35^ and streamflow observations at catchment scale^66^, respectively. The *E*_GLEAM_ and *E*_CSIRO_ are the same data sets used to derive the ${f_{{E_{\rm{i}}}}}$, which are estimated from global multiple satellite products and climate forcing data sets. The *E*_MERRAa_ and *E*_MERRAs_ are assimilation and land simulation data of the NASA’s Modern-Era Retrospective Analysis for Research and Applications (MERRA) products^67, 68^, respectively. The *E*_ERA_ is the assimilation data of ERA-Interim product^69^.

### Validation of the WUE model using eddy flux observations

The FLUXNET2015 data set is applied to validate the analytical WUE model. The FLUXNET2015 data set is the latest collection of global eddy flux observations, which includes sites of multiple regional flux networks and several improvements to the data quality control protocols and data processing pipeline. After latest update on July of 2016, FLUXNET2015 has 165 stations globally with observations up to 2014. All the monthly data of the 165 stations are collected firstly and some selection criteria are applied on some interested variables to screen data for validation.

Observed ecosystem annual WUE is estimated as the ratio of annual total GPP (in g C m^−2^ per year) to total evapotranspiration (*E* in mm per year) according to the definition of annual WUE in this study. Ecosystem annual GPP is estimated as the average of different estimates of annual GPP in the FLUXNET2015 data set using different methods partitioning of carbon flux (see http://fluxnet.fluxdata.org/data/fluxnet2015-dataset/data-processing/). Evapotranspiration is converted from observed latent heat flux (LE) from unit of W m^−2^ to the unit in equivalent depth of water in mm per year based on site observed monthly mean daily air temperature (*T*_a_). We used corrected LE data for estimating ecosystem annual WUE. Correction of LE is necessary as lack of energy closure is quite common in observed latent fluxes using eddy covariance techniques. The corrected LE data are provided in the FLUXNET2015 data set, which is based on the energy balance closure ratio and keep the Bowen ratio unchanged.

Criteria applied to select GPP and corrected LE data for estimating observed annual WUE include: (1) based on the data quality information in the FLUXNET2015 data set, site-year with >80% of high-quality observed or gap-filled data of hourly GPP and LE; (2) at least one pair of annual corrected LE and GPP are available; (3) energy closure bias is within 20%; (4) annual total GPP (in g C m^−2^ per year) and LE (in mm per year) are >10; and (5) site is not irrigated or wetlands. There are about 1180 site-years of data available in the FLUXNET2015 data set, of which about 47% and 20% satisfies the first two and all five selection criteria, respectively.

The input data for estimate ecosystem annual WUE at flux sites are the same as that for the global estimation. However, there is only one of the three collected global climate forcing data set is up to 2014 (i.e. CRU-NCEP data set). The method to estimate annual WUE at the site level using monthly data is the same as that applied at the global scale. To account the uncertainty in the estimated WUE, global leaf area index (*L*) and interception ratio (${f_{{E_{\rm{i}}}}}$) data sets at its original resolution and half degree resolution are used to estimate WUE at site level. At last, 51 sites and 229 site-years data are selected for validating the analytical ecosystem WUE model considering both selection criteria and available of other variables for estimating site level WUE. The list of sites and their key information are provided in Supplementary Table 3.

The validity of the analytical ecosystem WUE model is assessed using all the observed annual WUE, i.e. 229 station-years, and using annual trends of 11 sites with minimum length of 7 years of observed data. Sites used for trend validation are also listed in Supplementary Table 3. The first validation can be considered as a spatial validation of capability of the model to produce the variability of WUE between-sites. The second validation can be considered as a temporal validation of capability of the model to reproduce observed inter-annual (between-years) variability. In addition, the proposed method for estimating GPP is also validated at the site level and shown in Supplementary Fig. 9 (see Supplementary Discussion).

### Validation of estimated global WUE and GPP

The spatial patterns and magnitudes of estimated global WUE and GPP are compared against other independent estimates including one estimate using the MTE method^31^ and other six estimates by land surface models (Supplementary Table 4) of TRENDY modelling experiments^70^.

### Trend and its attribution

Trends in global WUE, *E* and GPP are estimated at both global and grid levels using the Mann–Kendall non-parametric trend test with Sen’s method, and significance levels on the basis of the Mann–Kendall tests.

The contributions of *E* and WUE to the total trend in GPP are isolated with three modelling experiments. One is called real modelling experiment with all the input data is the same as observed. The other two are control modelling experiments, which fixed only one contributing variable (i.e. *E* or WUE) at the initial condition in each modelling experiment. The trend of the differences between the real and control experiments is considered as the contribution of controlled variable to the total changes. Then, the contributions of *E* and WUE to total trends are isolated. Similarly, contributions of four variables (i.e. *C*_a_, *L*, *D* and ${f_{{E_{\rm{i}}}}}$) to the total trends in WUE are isolated with five modelling experiments. See Supplementary Tables 5 and 6 for more details.

### Data availability

All data used in this study are obtained from the literature or publically available databases, which can be found with the references provided in the section of estimating of ecosystem WUE and GPP and also in Supplementary Tables 1–4.

## Electronic supplementary material

Peer Review File

Supplementary Information
