# Supplementary material for: Recent increases in terrestrial carbon uptake at little cost to the water cycle
Source: Nat Commun. 2017 Jul 24;8:110. doi: 10.1038/s41467-017-00114-5 (PMC5524649; doi:10.1038/s41467-017-00114-5)
Supplement: Supplementary file 2 — Supplementary Information [file 41467_2017_114_MOESM2_ESM.pdf]

File name: Supplementary Information
Description: Supplementary Figures, Supplementary Tables, Supplementary Discussion and Supplementary References

File name: Peer Review File
Description:

Supplementary Discussion

We test the influences of different schemes for mapping global g_1 on the estimated water use efficiency (*WUE*). Mapping of global patterns of g_1 depends on g_1 values of different PFTs and global land cover map. In ref. 1, parameter g_1 is predicted to changes with moisture index and temperature as well. This alternative method for mapping g_1 is also considered. Comparing with the global g_1 map generated using the method in section 2.1, the alternative method produces much smoother changes in both g_1 values and spatial patterns, but there is no significant difference in estimated global *WUE* and its trend. Finally, the alternative method for mapping global map of g_1 is not adopted in this study for three reasons (1) the relationship of g_1 with moisture index and temperature is not very robust (see ref. 1); (2) no significant differences are found; and (3) is to keep a parsimonious parameterization of the *WUE* model.

Furthermore, two other different global land cover maps are collected to test the influences of different land cover on the estimated *WUE*. The two land cover maps are MODIS land cover map and the vegetation cover map used for the CABLE land surface model (see ref. 2). Results show that there is no significant difference in estimated global *WUE* patterns, magnitude and trends globally from three different land cover maps, but there are significant differences in some limited regions. Finally, the global synergetic land cover product, i.e. SYNMAP, is adopted as its overall advantages for carbon cycle modelling over other land cover products (see ref. 3). Analysis of the influences of different schemes for mapping global g_1 suggests that the importance of future global vegetation products to take account of the functional traits of vegetation for modelling of carbon and water cycles.

Estimated global mean annual gross primary productivity (*GPP*) has relative large standard deviation (146.1±21.3 Pg C year^{-1}), which is resulted from differences in the input data for variable vapour pressure deficit (*D*) (via *WUE*) and seven different evapotranspiration (*E*) datasets. The *WUE* model is very sensitive to variable *D*. Small differences in *D* from three different climate forcing datasets result in significant difference in estimated *WUE*. The mean annual *WUE* of three different input for *D* from the CRU-NCEP, PGF and WATCH climate forcing datasets are 1.64±0.02,

2.50±0.02, and 2.09±0.03 g C mm^{-1} H_2O from 1982 to 2011, and lead to significant differences in estimated mean annual *GPP* of 120.8±8.7, 168.0±10.1, and 149.5±8.7 Pg C $year^{-1}$, respectively. For different *E* datasets, three reanalysis datasets have a much larger mean annual *E* (650 mm $year^{-1}$) than the other four diagnostic *E* datasets (570 mm $year^{-1}$, see Supplementary Figure 3), which leads to significant variations in the estimated mean annual *GPP* as well but are smaller than those resulted from different inputs for variable *D*.

Differences in the *E* and *D* do not lead to any significant differences in the trends in both *WUE* and *GPP* and conclusion of this study, which can be observed from the small standard deviation of the trends from an ensemble of 12 *WUE* estimates and an ensemble of 84 estimates of *GPP* (see Figure 2).

From Figure 2c in the article, we can find that contributions of both *E* and *WUE* to the trend in estimated *GPP* have noticeable uncertainties comparing one standard deviations with the mean (i.e. error-bars and bars, respectively). The noticeable uncertainty in the contribution of *E* largely results from two of the seven *E* datasets, i.e. $E_{WB\text{-}MTE}$[4] and E_{MARRAs}[5]. Amongst the seven *E* datasets, the $E_{WB\text{-}MTE}$ has the largest trend, while the E_{MARRAs} has the smallest trend and is the only one has negative trend in *E* (see Supplementary Figure 3). Largest trend in the $E_{WB\text{-}MTE}$ is potentially resulted from some disadvantages of the methodology of this dataset (see ref. 4). If these two *E* datasets are excluded, trend in *GPP* and contribution from *E* are 0.83±0.05, and 0.09±0.04 Pg C $year^{-2}$, respectively, with which the conclusion of this study is the same but the uncertainty in the estimated *GPP* trend and contribution from *E* are significantly reduced.

The noticeable uncertainty in the contributions of *WUE* to the estimated trend in global *GPP* largely results from the contribution from leaf area index (*L*) (see Figure 2d), which is dominated by a step change before and after 2000 in the current version of GLASS LAI dataset, particularly in the tropical region (see ref. 6). The estimated *WUE* trend from the GLASS LAI product is significantly larger (17.5±2.9 mg C mm^{-1} H_2O $year^{-1}$) than that from the GIMMS LAI product (10.0±1.6 mg C mm^{-1} H_2O $year^{-1}$). The contribution of LAI to the total *WUE* trend derived from the GLASS LAI product is significantly larger (11.5±2.1 mg C mm^{-1} H_2O $year^{-1}$) than that from the GIMMS LAI

product (3.4±0.6 mg C mm^{-1} H_2O year^{-1}) as well. Both LAI datasets lead to same conclusion that increase in global *L* is an important contributor to changes in *WUE*. However, the inconsistency in the GLASS LAI product has led to a larger role of LAI for the trend in global *WUE* and *GPP*. Importantly, our results highlight the importance of the response of *L* to environmental changes for predicting future changes in water and carbon cycles.

Another two options for defining the growing season are also evaluated, one is with a threshold monthly mean temperature of 5 C, and the other one is the global phenology product derived from MODIS data[7, 8]. Results show that conclusions of these two growing season options remain the same as these derived using a threshold temperature of 0 C. The threshold temperature of 0 C for the growing season is adopted in this study as this measure minimizes the influence of non-growing season soil evaporation and thus to keep a parsimonious parameterization of the *WUE* equation.

According to our proposed *WUE* model, the influence of leaf area index (*L*) on ecosystem *WUE* is accounted in the second and third terms of the equation (6), i.e. $[1 - exp(-kL)]$ and $\left(1 - f_{E_\mathrm{i}}\right)$, respectively. The second term suggests that ecosystem *WUE* increases with *L*, but the third term implies that ecosystem *WUE* decreases with *L* as generally interception ratio is positively related to *L*[9].

Essentially, the second and third terms together represent the fraction of transpiration (E_t) to total evaporation (see equation (3)), i.e. transpiration ratio (denoted as f_{E_t}). To demonstrate the control of *L* on ecosystem *WUE*, we plot the relationship between mean annual *L* and f_{E_t} based on the GIMMS LAI3g dataset and interception ratio data from E_{GLEAM} as shown in Supplementary Figure 8.

On average, the blue line in the Supplementary Figure 8 reflects the control of *L* on the estimated ecosystem *WUE*, which suggests that ecosystem *WUE* increases with *L* to about 3 and then decreases with *L* at the global scale.

Annual *GPP* at the site level is estimated using the WEC method (i.e. equation (7)) and validated against the site observed *GPP*. The Supplementary Figure 9 shows the validation of site *GPP* using all the 229 station-years. The linear correlation coefficient

(*r*) between observed and estimated annual *GPP* is about 0.76, with Nash-Sutcliffe model efficiency (NSE[10]) of 0.47, root mean squared error (RMSE) of 487.3 g C m^{-2} year^{-1}, mean error (ME) of −152.0 g C m^{-2} year^{-1} and relative error (RE) of −11.5%. The slope of the regressed line between observed and estimated annual *WUE* passing through the origin is 0.86, with an adjusted R^2 of 0.89.

Estimated trends in *GPP* at global scale and also in 17 different eco-regions are compared with six LSMs (see Supplementary Table 4). For comparing our estimates with other studies, terrestrial vegetated area are grouped into 17 different ecoregions in terms of major ecoregions delineated by ref. 11 (also see http://www.worldwildlife.org/publications/terrestrial-ecoregions-of-the-world) and continental boundaries. The details of the ecoregions are shown in the Supplementary Table 7 and maps are shown in the Supplementary Figure 6. Across 17 different ecoregions, Supplementary Figure 7 shows that trends in *GPP* estimated by the WEC method fall within the ranges derived from six LSMs, except the temperate forest in north American (TempF-NAM) and boreal forest (BF) regions, where the WEC method estimated a larger increase rate in *GPP* than that derived from LSMs.

For the trend in *GPP* at the global scale, the WEC method estimates a higher mean value and wider range of increase rate in global *GPP* than that derived from six LSMs over the same period and other reported values. Results from the WEC method with an ensemble of 84 estimates show that global *GPP* has increased about 0.83±0.26 Pg C year^{-2} over 1982-2011 with a range of 0.33 ~ 1.30 Pg C year^{-2}. Based on the six LSMs from TrendyV3 modelling experiment [12], global *GPP* has increased about 0.44±0.08 Pg C year^{-2} with a range of 0.32 ~ 0.57 Pg C year^{-2} over 1982 – 2011. Other independent studies reported that global *GPP* has increased from 0.2 to 0.66 Pg C year^{-2} during the past one or two decades (see ref. 13, ref. 14 and ref. 15). Basically, the WEC method estimated a larger increase in global *GPP* in the past three decades than other modelling results, which could be partially resulted from uncertainty in one of the leaf area index products (see Supplementary Discussion on the influences of *L* on estimated results). Based on the GIMMS LAI3g leaf area index product only, estimated global *GPP* trend by the WEC method is 0.59±0.12 Pg C year^{-2} (0.33 ~ 0.87 Pg C year^{-2}), which is very close to that derived from six LSMs and to other independent studies.

Supplementary Table 1 | g_1 value of different plant function types compiled by Lin et al., 2015.

NO.	Vegetation type	PFT	g_1 value
1	C4 grass	C4 grass/crop	1.62
2	Evergreen gymnosperms tree	Evergreen needle leaf forest (ENF)	2.35
3	Deciduous savanna tree	Deciduous broadleaf forest (DBF) - savanna	2.98
4	Evergreen angiosperms tree	Evergreen broadleaf forest (EBF)	3.37
5	Tropical rainforest tree	EBF - tropical rainforest	3.77
6	Shrub	Shrub	4.22
7	C3 grass	C3 grass	4.50
8	Deciduous angiosperms tree	DBF	4.64
9	C3 crop	C3 crop	5.79
10	Evergreen savanna tree	EBF - savanna	7.18

Supplementary Table 2 I Summary of global evapotranspiration (*E*) datasets used for estimation of ecosystem gross primary production (*GPP*).

Name	research group	references
E_{MTE}	Max Planck Institute, Germany	ref. 16; ref. 17
E_{GLEAM}	VU University, The Netherlands	ref. 18; ref. 19
E_{CSIRO}	CSIRO Land and Water, Australia	ref. 20
$E_{WB\text{-}MTE}$	Peking University	ref. 4
E_{MERRAa}	Goddard Space Flight Centre of NASA	ref. 21
E_{MERRAs}	Goddard Space Flight Centre of NASA	ref. 5
E_{ERA}	European Centre for Medium-Range Weather Forecasts	ref. 22

Supplementary Table 3 I The basic information of the flux sites used for model validation

NO.	Code	Site Name	Latitude (degree N)	Longitude (degree E)	Elevation (m)	IGBP	g_1
1	AT-Neu	Neustift	47.12	11.32	976	GRA	2.9
2	AU-Cpr	Calperum	-34.00	140.59	62	SAV	4.8
3	AU-DaP	Daly River Savanna	-14.06	131.32	71	GRA	4.2
4	AU-DaS	Daly River Cleared	-14.16	131.39	75	SAV	4.2
5	AU-Dry	Dry River	-15.26	132.37	176	SAV	4.1
6	AU-RDF	Red Dirt Melon Farm, Northern Territory	-14.56	132.48	188	WSA	4.1
**7**	**AU-Tum**	**Tumbarumba**	-35.66	148.15	1259	EBF	4.1
8	AU-Whr	Whroo	-36.67	145.03	151	EBF	5.5
9	BE-Bra	Brasschaat	51.31	4.52	17	MF	5.5
10	BE-Vie	Vielsalm	50.31	6.00	492	MF	4.6
11	BR-Sa3	Santarem-Km83-Logged Forest	-3.02	-54.97	181	EBF	4.1
12	CA-TP1	Ontario - Turkey Point 2002 Plantation White Pine	42.66	-80.56	200	ENF	5.8
13	CH-Cha	Chamau	47.21	8.41	394	GRA	4.8
14	CH-Fru	Fruebuel	47.12	8.54	982	GRA	4.2
15	CN-Cng	Changling	44.59	123.51	142	GRA	3.8
16	DE-Hai	Hainich	51.08	10.45	464	DBF	5.5
17	DE-Lkb	Lackenberg	49.10	13.30	1302	ENF	3.9
18	DE-Obe	Oberbärenburg	50.78	13.72	782	ENF	5.1
**19**	**DE-Tha**	**Tharandt**	50.96	13.57	386	ENF	5.1
20	DK-Sor	Soroe	55.49	11.64	46	DBF	5.7
21	ES-LJu	Llano de los Juanes	36.93	-2.75	1616	OSH	4.4
22	FI-Hyy	Hyytiala	61.85	24.30	180	ENF	2.4
**23**	**FR-Gri**	**Grignon**	48.84	1.95	122	CRO	5.6
24	IT-CA3	Castel d'Asso 3	42.38	12.02	199	DBF	4.7
**25**	**IT-Lav**	**Lavarone**	45.96	11.28	1352	ENF	4.5
26	IT-Noe	Arca di Noé - Le Prigionette	40.61	8.15	27	CSH	4.6
27	IT-Ren	Renon	46.59	11.43	1737	ENF	3.3
28	IT-Ro2	Roccarespampani 2	42.39	11.92	173	DBF	4.9
**29**	**MY-PSO**	**Pasoh Forest Reserve (PSO)**	2.97	102.31	147	EBF	4.6
30	NL-Hor	Horstermeer	52.24	5.07	-2	GRA	5.4
31	NL-Loo	Loobos	52.17	5.74	34	ENF	4.7
32	RU-Fyo	Fyodorovskoye	56.46	32.92	275	ENF	3.5
33	SD-Dem	Demokeya	13.28	30.48	537	SAV	3.8
34	US-AR1	ARM USDA UNL OSU Woodward Switchgrass 1	36.43	-99.42	613	GRA	3.4

35	US-AR2	ARM USDA UNL OSU Woodward Switchgrass 2	36.64	-99.60	647	GRA	3.6
**36**	**US-ARM**	**ARM Southern Great Plains site- Lamont**	36.61	-97.49	316	CRO	3.8
37	US-Blo	Blodgett Forest	38.90	-120.63	1329	ENF	2.6
38	US-Goo	Goodwin Creek	34.25	-89.87	98	GRA	4.2
39	US-KS2	Kennedy Space Center (scrub oak)	28.61	-80.67	2	CSH	2.9
**40**	**US-Me2**	**Metolius-intermediate aged ponderosa pine**	44.45	-121.56	1254	ENF	2.5
41	US-Ne3	Mead - rainfed maize-soybean rotation site	41.18	-96.44	361	CRO	4.0
42	US-Oho	Oak Openings	41.55	-83.84	204	DBF	4.7
43	US-SRG	Santa Rita Grassland	31.79	-110.83	1291.6	GRA	3.7
**44**	**US-SRM**	**Santa Rita Mesquite**	31.82	-110.87	1119.4	WSA	3.7
45	US-Ton	Tonzi Ranch	38.43	-120.97	168.7	WSA	3.3
46	US-Tw3	Twitchell Alfalfa	38.12	-121.65	-2.6	CRO	4.7
**47**	**US-Var**	**Vaira Ranch- Ione**	38.41	-120.95	161.7	GRA	3.3
48	US-Whs	Walnut Gulch Lucky Hills Shrub	31.74	-110.05	1373.7	OSH	3.9
**49**	**US-Wkg**	**Walnut Gulch Kendall Grasslands**	31.74	-109.94	1527.6	GRA	3.9
**50**	**ZA-Kru**	**Skukuza**	-25.02	31.50	361.5	SAV	3.9
51	ZM-Mon	Mongu	-15.44	23.25	1087.1	DBF	4.0

Notes: data of sites used for trend validation are highlighted with bold font. Column IGBP is the vegetation type identified from IGBP land cover map and the abbreviations of different land cover types please refer to http://www.fluxdata.org/DataInfo/Dataset%20Doc%20Lib/VegTypeIGBP.aspx and the definitions in the IGBP dataset (http://glcf.umd.edu/data/lc/).

Supplementary Table 4 I Summary of land surface models used in this study.

No.	Model	full name and Website	references
1	CABLE	Community Atmosphere-Biosphere-Land Exchange model https://trac.nci.org.au/trac/cable	ref. 23; ref. 2
2	CLM	Community Land Model http://www.cgd.ucar.edu/tss/clm/	ref. 24
3	ISAM	Integrated Science Assessment Model http://climate.atmos.uiuc.edu/isam2/	ref. 25
4	JULES	Joint UK Land Environment Simulator https://jules.jchmr.org/	ref. 26; ref. 27
5	ORCHIDEE	Organising Carbon and Hydrology In Dynamic Ecosystems http://labex.ipsl.fr/orchidee/	ref. 28
6	VISIT	Vegetation Integrative SImulation Tool	ref. 29

Supplementary Table 5 I Modelling experiments for isolating the contribution of evapotranspiration (*E*) and water use efficiency (*WUE*) total trend in global ecosystem gross primary production (*GPP*)

No.	*E*	*WUE*	Descriptions
Expt1	N	N	Experiment 1 (Expt1) is considered as a "real" estimation. Results are used to estimate total trends in global ecosystem *GPP* and as references for other two experiments to isolate the contributions of *E* and *WUE* to the total trend.
Expt2	**Y**	N	The same as Experiment 1 (Expt1), except that *E* is kept as initial condition. Experiment 2 (Expt2) is designed to isolate the contribution of changes in *E* to global trends in *GPP* in terms of the differences between Expt1 and Expt2.
Expt3	N	**Y**	The same as Experiment 1 (Expt1), except that *WUE* is kept as initial condition. Experiment 3 (Expt3) is designed to isolate the contribution of changes in *WUE* to global trends in *GPP* in terms of the differences between Expt1 and Expt3.

Note: "Y" and "N" indicate whether input data for the modelling experiments is fixed at initial conditions or not, i.e. values of the beginning year.

Supplementary Table 6 I Modelling experiments for isolating the contribution of different factors to total trend in global ecosystem water use efficiency (*WUE*)

No.	Input data				Descriptions
	C_a	D	L	f_{Ei}	
Expt1	N	N	N	N	Experiment 1 (Expt1) is considered as a "real" estimation. Results are used to estimate total trends in global ecosystem *WUE* and as references for other modelling experiments to isolate the contributions of different factors to the total trend.
Expt2	**Y**	N	N	N	The same as Experiment 1 (Expt1), except that C_a is kept at initial condition. Experiment 2 (Expt2) is designed to isolate the contribution of changes in C_a to global trends in ecosystem *WUE* in terms of the differences between Expt1 and Expt2.
Expt3	N	**Y**	N	N	The same as Experiment 1 (Expt1), except the D is kept at the initial condition. Experiment 3 (Expt3) is designed to isolate the contribution of changes in D to global trends in ecosystem *WUE* in terms of the differences between Expt1 and Expt3.
Expt4	N	N	**Y**	N	The same as Experiment 1 (Expt1), except the L is kept at initial condition. Experiment 4 (Expt4) is designed to isolate the contribution of changes in L to global trends in ecosystem *WUE* in terms of differences between Expt1 and Expt4.
Expt5	N	N	N	**Y**	The same as Experiment 1 (Expt1), except the fraction of interception (f_{Ei}) is kept at the initial condition. Experiment 5 (Expt5) is designed to isolate the contribution of changes in f_{Ei} to global trends in ecosystem *WUE* in terms of differences between Expt1 and Expt5.

Note: "Y" and "N" indicate whether input data for the modelling experiments is fixed at initial conditions or not, i.e. values of the beginning year.

Supplementary Table 7 | List of global ecoregions

NO.	Major ecoregions	Continental regions	Abbreviations
1	Tropical forest	American	TropF-AM
2		African	TropF-AF
3		Asian	TropF-AS
4	Temperate forest	North American	TempF-NAM
5		European	TempF-EU
6		Asian	TempF-AS
7		Southern Hemisphere	TempF-SH
8	Boreal forest	North American	BF-NAM
9		Eurasian	BF-EA
10	Tropical grass, savanna and shrub	American	TropGSS-AM
11		African	TropGSS-AF
12		Australia	TropGSS-AU
13	Temperate grass, savanna and shrub	North American	TempGSS-NAM
14		Eurasian	TempGSS-EA
15		Southern Hemisphere	TempGSS-SH
16	Tundra	Tundra	Tundra
17	Arid	Arid	Arid

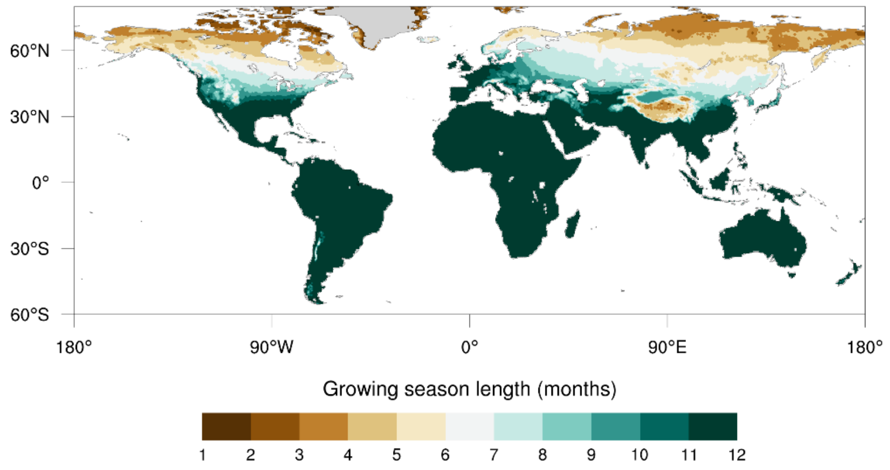

Supplementary Figure 1 | Global pattern of mean growing season length in months.

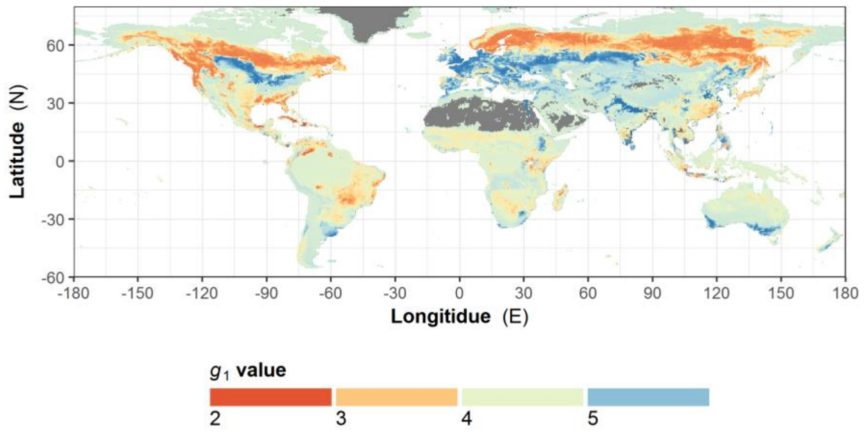

Supplementary Figure 2 | Spatial details of estimated global parameter g_1.

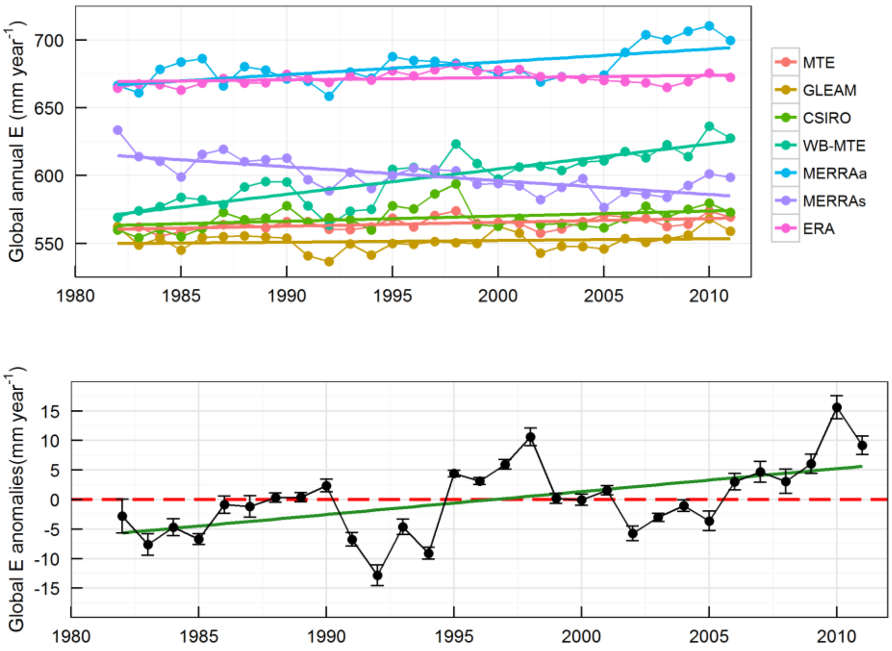

Supplementary Figure 3 I Global annual evapotranspiration (*E*) and its anomalies of the seven datasets. The upper panel is the global annual *E* of the seven datasets. The straight lines are the linear trends of each datasets. The lower panel is the ensemble mean annual anomalies of the seven datasets. The error bar shows the standard error of the seven datasets.

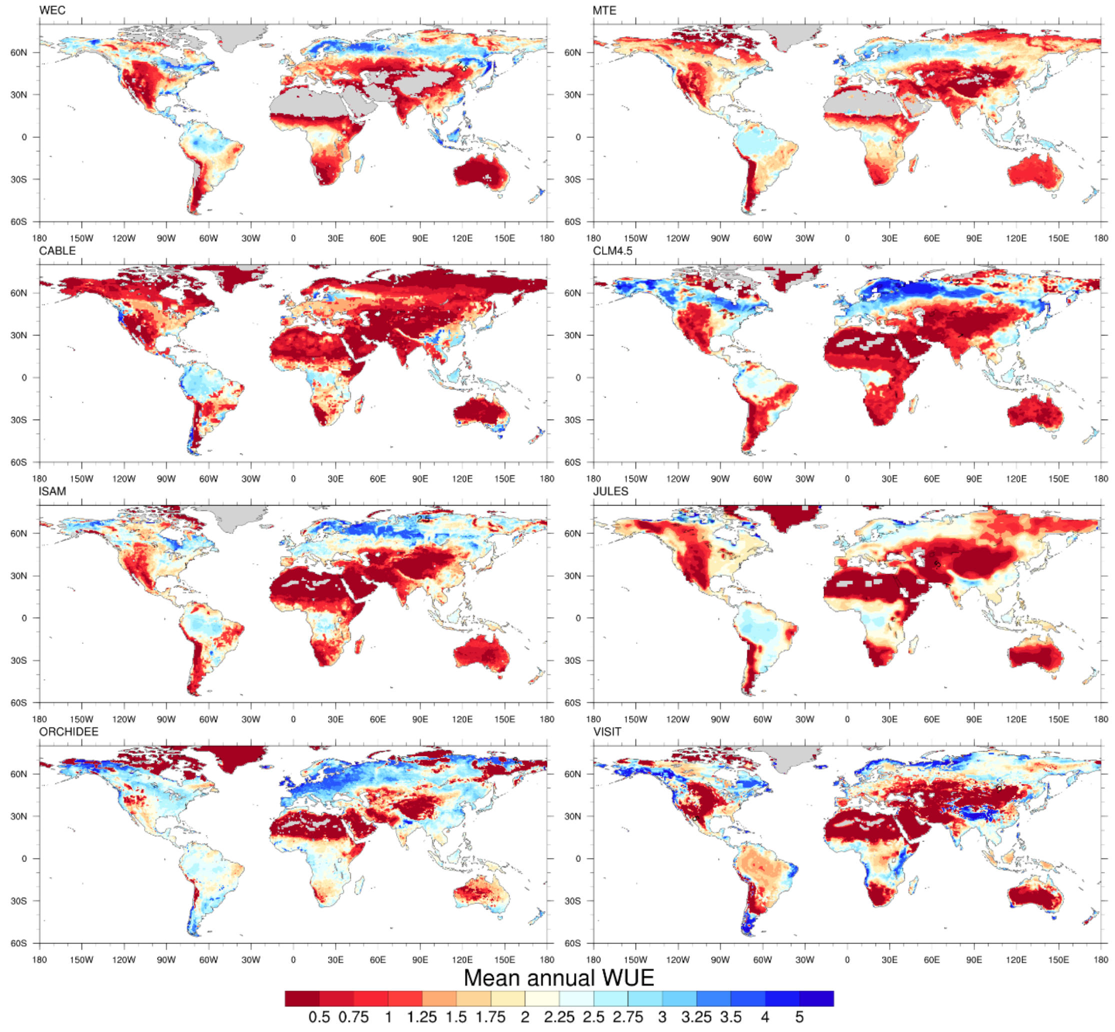

Supplementary Figure 4 | Comparison of the spatial details of global mean annual water use efficiency (*WUE*) over 1982–2011. Estimated *WUE* (in g C mm^{-1} H_2O) from the analytical method is compared with the model tree ensemble (MTE) estimate and that from six land surface models.

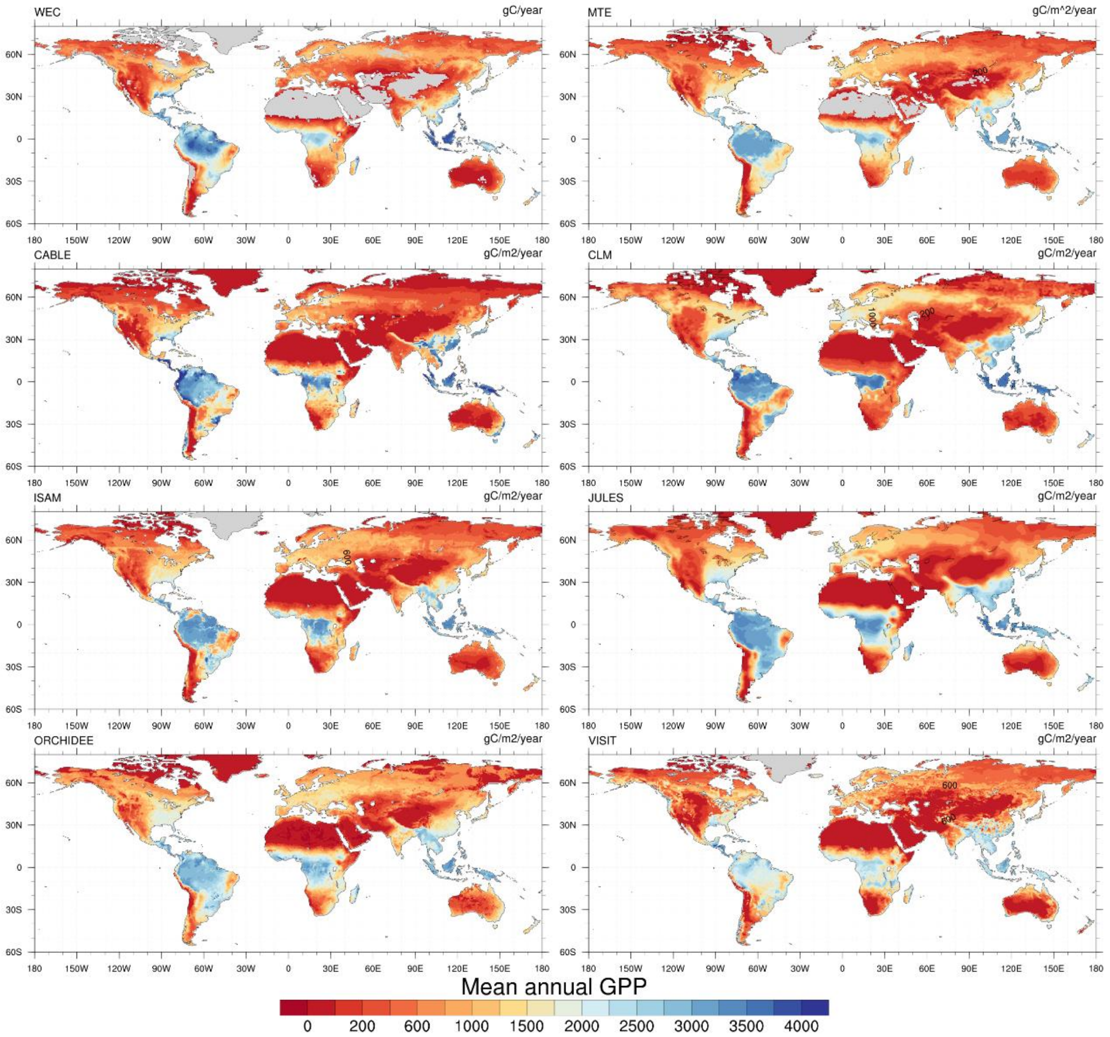

Supplementary Figure 5 | Comparison of the spatial details of global mean annual gross primary production (*GPP*) over 1982–2011. Estimated *GPP* (in g C m^{-2} $year^{-1}$) from the analytical method is compared with the model tree ensemble (MTE) estimate and that from six land surface models.

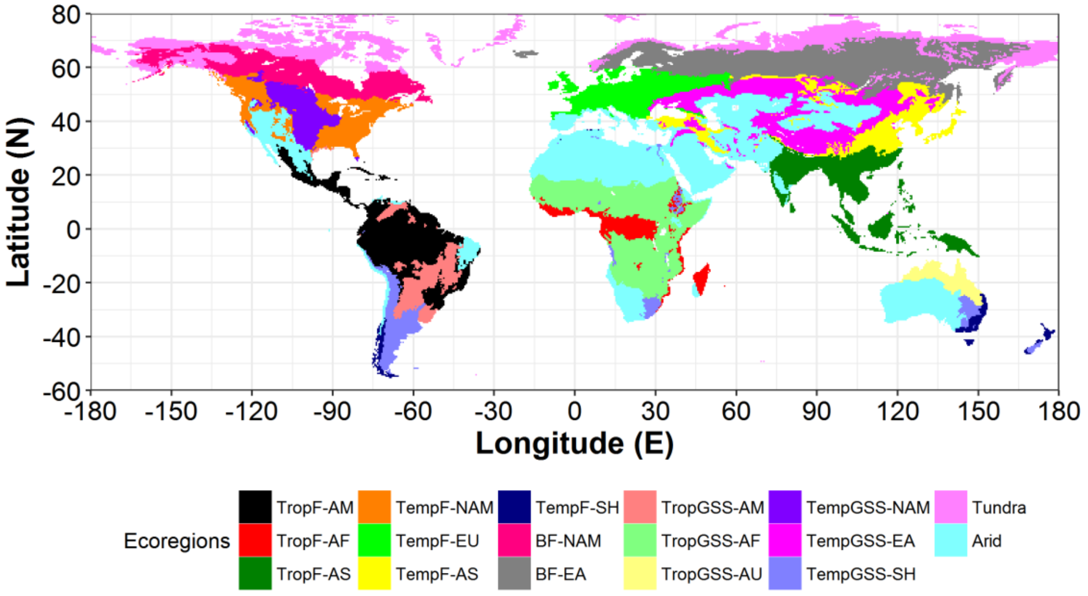

Supplementary Figure 6 | Global ecoregions. The name of ecoregions are provided in Supplementary Table 7.

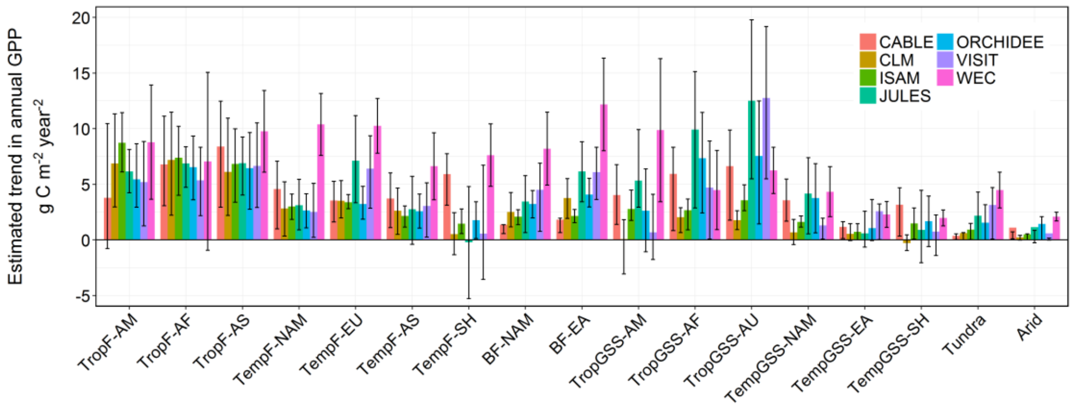

Supplementary Figure 7 | Comparison of estimated trends in ecosystem gross primary production (*GPP*) with 6 land surface models (LSMs) in 17 different eco-regions over the period of 1982-2011. The ecoregions are defined in the Supplementary Table 7 and shown in the Supplementary Figure 6. The bars represent of the mean trends of all the grid cells within different ecoregions. The error-bars show the inter-quantile range of different LSMs and one standard deviation of 84 ensembles within different ecoregions, respectively.

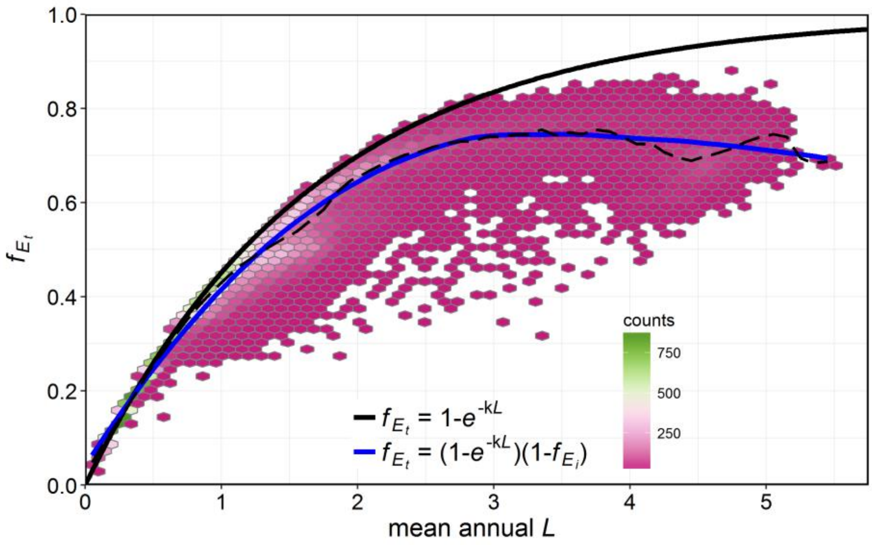

Supplementary Figure 8 | The relationship between ecosystem transpiration ratio (f_{E_t}) and leaf area index (L). Hexagon binning plot showing relationship between mean annual L and $f_{E_t}\left(=[1-exp(-kL)](1-f_{E_i})\right)$ over all the vegetated land cells. The colour of the hexagon indicates the number of land cells. The broken black line is the median of f_{E_t} by binning mean annual L with a step of 0.1, and the blue line is smoothed median f_{E_t} (i.e. broken line) using non-parametric local regression method (i.e. LOESS). The solid black line shows the relationship between f_{E_t} and L by neglecting the interception ratio, i.e. $f_{E_t}=[1-exp(-kL)]$.

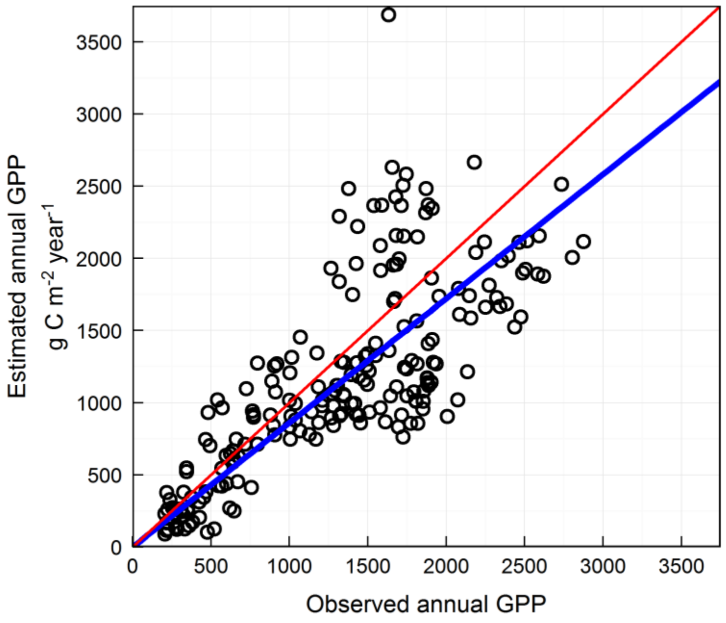

Supplementary Figure 9 | Validation of estimated site gross primary production (*GPP*) using the proposed method in this study. The red line is the 1:1 line and blue line is fitted using least square regression method.

Supplementary References

[1]Lin, Y., Medlyn, B. E., Duursma, R. A., Prentice, I. C., Wang, H., and Baig, S. *et al.* Optimal stomatal behaviour around the world, *Nature Clim. Change* **5**, 459-464 (2015).

[2]Wang, Y. P., Kowalczyk, E., Leuning, R., Abramowitz, G., Raupach, M. R., and Pak, B. *et al.* Diagnosing errors in a land surface model (CABLE) in the time and frequency domains, *J. Geophys. Res. Biogeosci.* **116**, G01034 (2011).

[3]Jung, M., Henkel, K., Herold, M., and Churkina, G. Exploiting synergies of global land cover products for carbon cycle modeling, *Remote Sens. Environ.* **101**, 534-553 (2006).

[4]Zeng, Z., Wang, T., Zhou, F., Ciais, P., Mao, J., and Shi, X. *et al.* A worldwide analysis of spatiotemporal changes in water balance-based evapotranspiration from 1982 to 2009, *J. Geophys. Res. Atmos.* **119**, 1186-1202 (2014).

[5]Reichle, R. H., Koster, R. D., De Lannoy, G. J. M., Forman, B. A., Liu, Q., and Mahanama, S. P. P. *et al.* Assessment and Enhancement of MERRA Land Surface Hydrology Estimates, *J. Climate* **24**, 6322-6338 (2011).

[6]Zhu, Z., Piao, S., Myneni, R. B., Huang, M., Zeng, Z., and Canadell, J. G. *et al.* Greening of the Earth and its drivers, *Nature Clim. Change* **6**, 791 – 795 (2016).

[7]Zhang, X., Friedl, M. A., and Schaaf, C. B. Global vegetation phenology from Moderate Resolution Imaging Spectroradiometer (MODIS): Evaluation of global patterns and comparison with in situ measurements, *J. Geophys. Res. Biogeosci.* **111**, G04017 (2006).

[8]Zhang, X., Friedl, M. A., Schaaf, C. B., Strahler, A. H., and Liu, Z. Monitoring the response of vegetation phenology to precipitation in Africa by coupling MODIS and TRMM instruments, *J. Geophys. Res. Atmos.* **110**, D12103 (2005).

[9]Miralles, D. G., Gash, J. H., Holmes, T. R. H., de Jeu, R. A. M., and Dolman, A. J. Global canopy interception from satellite observations, *J. Geophys. Res. Atmos.* **115**, D16122 (2010).

[10]Nash, J. E., and Sutcliffe, J. V. River flow forecasting through conceptual models part I — A discussion of principles, *J. Hydrol.* **10**, 282-290 (1970).

[11]Olson, D. M., Dinerstein, E., Wikramanayake, E. D., Burgess, N. D., Powell, G. V. N., and Underwood, E. C. *et al.* Terrestrial Ecoregions of the World: A New Map of Life on Earth: A new global map of terrestrial ecoregions provides an innovative tool for conserving biodiversity, *BioScience* **51**, 933 -938 (2001).

[12]Le Quéré, C., Peters, G. P., Andres, R. J., Andrew, R. M., Boden, T. A., and Ciais, P. *et al.* Global carbon budget 2013, *Earth Syst. Sci. Data* **6**, 235-263 (2014).

[13]Anav, A., Friedlingstein, P., Kidston, M., Bopp, L., Ciais, P., and Cox, P. *et al.* Evaluating the Land and Ocean Components of the Global Carbon Cycle in the CMIP5 Earth System Models, *J. Climate* **26**, 6801-6843 (2013).

[14]Anav, A., Friedlingstein, P., Beer, C., Ciais, P., Harper, A., and Jones, C. *et al.* Spatiotemporal patterns of terrestrial gross primary production: A review, *Rev. Geophys.* **53**, 785-818 (2015).

[15]Jiang, C., and Ryu, Y. Multi-scale evaluation of global gross primary productivity and evapotranspiration products derived from Breathing Earth System Simulator (BESS), *Remote Sens. Environ.* **186**, 528-547 (2016).

[16]Jung, M., Reichstein, M., Ciais, P., Seneviratne, S. I., Sheffield, J., and Goulden, M. L. *et al.* Recent decline in the global land evapotranspiration trend due to limited moisture supply, *Nature* **467**, 951-954 (2010).

[17]Jung, M., Reichstein, M., Margolis, H. A., Cescatti, A., Richardson, A. D., and Arain, M. A. *et al.* Global patterns of land-atmosphere fluxes of carbon dioxide, latent heat, and sensible heat derived from eddy covariance, satellite, and meteorological observations, *J. Geophys. Res.* **116**, G00J07 (2011).

[18]Miralles, D. G., Holmes, T. R. H., De Jeu, R. A. M., Gash, J. H., Meesters, A. G. C. A., and Dolman, A. J. Global land-surface evaporation estimated from satellite-based observations, *Hydrol. Earth Syst. Sc.* **15**, 453 (2011).

[19]Miralles, D. G., van den Berg, M. J., Gash, J. H., Parinussa, R. M., de Jeu, R. A. M., and Beck, H. E. *et al.* El Niño – La Niña cycle and recent trends in continental evaporation, *Nature Clim. Change* **4**, 122-126 (2014).

[20]Zhang, Y., Peña-Arancibia, J. L., McVicar, T. R., Chiew, F. H. S., Vaze, J., and Liu, C. *et al.* Multi-decadal trends in global terrestrial evapotranspiration and its components, *Sci. Rep.-UK* **6**, 19124 (2016).

[21]Rienecker, M. M., Suarez, M. J., Gelaro, R., Todling, R., Bacmeister, J., and Liu, E. *et al.* MERRA: NASA's Modern-Era Retrospective Analysis for Research and Applications, *J. Climate* **24**, 3624-3648 (2011).

[22]Dee, D. P., Uppala, S. M., Simmons, A. J., Berrisford, P., Poli, P., and Kobayashi, S. *et al.* The ERA-Interim reanalysis: configuration and performance of the data assimilation system, *Q. J. Roy. Meteor. Soc.* **137**, 553-597 (2011).

[23]Kowalczyk, E. A., Wang, Y. P., Law, R. M., Davies, H. L., McGregor, J. L., and Abramowitz, G. The CSIRO Atmosphere Biosphere Land Exchange (CABLE) model for use in climate models and as an offline model, 37 pp, CSIRO Marine and Atmospheric Research, Aspendale, Victoria (2006).

[24]Oleson, K. W., Lawrence, D. M., Bonan, G. B., Flanner, M. G., Kluzek, E., and Lawrence, P. J. *et al.* Technical Description of version 4.0 of the Community Land Model (CLM), 266 pp (2010).

[25]Jain, A. K., and Bach, W. The effectiveness of measures to reduce the man-made greenhouse effect. The application of a Climate-policy Model, *Theor. Appl. Climatol.* **49**, 103-118.

[26]Best, M. J., Pryor, M., Clark, D. B., Rooney, G. G., Essery, R. L. H., and Ménard, C. B. *et al.* The Joint UK Land Environment Simulator (JULES), model description – Part 1: Energy and water fluxes, *Geosci. Model Dev.* **4**, 677-699 (2011).

[27]Clark, D. B., Mercado, L. M., Sitch, S., Jones, C. D., Gedney, N., and Best, M. J. *et al.* The Joint UK Land Environment Simulator (JULES), model description – Part 2: Carbon fluxes and vegetation dynamics, *Geosci. Model Dev.* **4**, 701-722 (2011).

[28]Krinner, G., Viovy, N., de Noblet-Ducoudré, N., Ogée, J., Polcher, J., and Friedlingstein, P. *et al.* A dynamic global vegetation model for studies of the coupled atmosphere-biosphere system, *Global Biogeochem. Cy.* **19**, GB1015 (2005).

[29]Kato, E., Kawamiya, M., Kinoshita, T., and Ito, A. Development of spatially explicit emission scenario from land-use change and biomass burning for the input data of climate projection, *Procedia Environmental Sciences* **6**, 146-152 (2011).
